# Solid-State Microwave Drying for Medical Cannabis Inflorescences: A Rapid and Controlled Alternative to Traditional Drying

**DOI:** 10.1089/can.2022.0051

**Published:** 2024-02-12

**Authors:** Almog Uziel, Looz Milay, Shiri Procaccia, Ronen Cohen, Amir Burstein, Liron Sulimani, Inbar Shreiber-Livne, Dan Lewitus, David Meiri

**Affiliations:** ^1^ The Laboratory of Cancer Biology and Cannabinoid Research, Faculty of Biology, Technion - Israel Institute of Technology, Haifa, Israel.; ^2^ The Russell Berrie Nanotechnology Institute, Technion - Israel Institute of Technology, Haifa, Israel.; ^3^ Cannasoul Analytics, Caesarea, Israel.; ^4^ Goji Research Ltd., Kefar Sava, Israel.; ^5^ Department of Polymer Materials Engineering, Shenkar College of Engineering, Design and Art, Ramat Gan, Israel.

**Keywords:** medical Cannabis, phytocannabinoids, terpenoids, drying technology, microwave

## Abstract

**Introduction:**

As the medical use of Cannabis is evolving there is a greater demand for high-quality products for patients. One of the main steps in the manufacturing process of medical Cannabis is drying. Most current drying methods in the Cannabis industry are relatively slow and inefficient processes.

**Materials and Methods:**

This article presents a drying method based on solid-state microwave (MW) that provides fast and uniform drying, and examines its efficiency for drying Cannabis inflorescences compared with the traditional drying method. We assessed 67 cannabinoids and 36 terpenoids in the plant in a range of drying temperatures (40°C, 50°C, 60°C, and 80°C). The identification and quantification of these secondary metabolites were done by chromatography methods.

**Results:**

This method resulted in a considerable reduction of drying time, from several days to a few hours. The multiple frequency-phase combination states of the system allowed control and prediction of moisture levels during drying, thus preventing overdrying. A drying temperature of 50°C provided the most effective results in terms of both short drying time and preservation of the composition of the secondary metabolites compared with traditional drying. At 50°C, the chemical profile of phytocannabinoids and terpenoids was best kept to that of the original plant before drying, suggesting less degradation by chemical reactions such as decarboxylation. The fast-drying time also reduced the susceptibility of the plant to microbial contamination.

**Conclusion:**

Our results support solid-state MW drying as an effective postharvest step to quickly dry the plant material for improved downstream processing with a minimal negative impact on product quality.

## Introduction

Throughout history, the Cannabis plant has been widely used for spiritual and recreational purposes, as well as for its broad therapeutic effects.^1^ As its medical use continues to evolve, on one hand, and an increasing number of states approve its administration on the other hand,^2^ a greater demand for effective high-quality products for patients is also emerging.

The Cannabis plant harbors >500 natural compounds from different chemical classes, some belong to primary metabolism, while others represent secondary metabolites.^1,3^ Among them are phytocannabinoids and terpenoids, secondary metabolites that are biosynthesized in the glandular trichomes of female inflorescences, to which the therapeutic effects of the plant have been mainly attributed.^4–6^ Cannabis chemovars differ significantly in their chemical composition, that is, the content and profile of these active metabolites. The chemical composition depends on a range of factors, including the genetics of the plant, growth conditions, harvest time, postharvest processing methods, and storage conditions and duration.^7–9^

Drying is the first critical step in the postharvest processing and preservation of Cannabis. It is the postharvest operation of removing the excess moisture from newly harvested plants to prevent chemical and microbiological degradation.^10,11^ It is also one of the rate-determining steps in Cannabis manufacturing.

Usually, traditional drying takes place under aeration in closed rooms with controlled temperature and humidity levels for at least 5–6 days^12^ and up to 2 weeks. Either the whole plant or the branches with inflorescences are hung upside down. As the bud dries, water from the stem slowly migrates into the bud, slowing the drying process. Another variation is “screen drying,” in which trimmed buds are placed on drying screens. In this method, the drying time is shorter due to the large effective surface area available for drying. However, it results in uneven drying as the size of the buds influences the drying rate. Fans, heaters, and dehumidifiers are sometimes used to speed up the drying process. The texture and crispness of the buds are used to determine whether the product is dried.^12,13^

Currently, there are no models to predict the drying end-point or the overall drying time. In addition to consuming excess energy, this slow process also increases the risk of mold growth and product spoilage since microbes tend to thrive in a humid and wet environment.^12,14–16^ The drying process may be followed by curing to balance the moisture content in the buds and age the dry Cannabis before consumption.^17^ This is suggested to reduce the content of starch, sugar, and chlorophyll and improve the resultant flavor. In a pharmaceutical crop, secondary metabolite content is the central focus and less importance is given to taste.^18^

From a technological perspective, standardizing and optimizing the drying process is part of the trend toward high-quality and chemically standardized Cannabis products.^11,19,20^ Alternative drying methods have been proposed in recent years to save time, reduce the risk of microbial load, and improve product uniformity and consistency.^12^ Among these are microwave (MW)-vacuum and radiofrequency (RF), which do not rely on conduction and convection to deliver thermal energy as in conventional drying.^21–23^ Rather, electromagnetic waves are transferred directly to the heated product, providing fast and volumetric heating.^24^ RFs range from 3 kHz to 300 GHz, with MW frequencies as a subset that includes frequencies from 300 MHz to 300 GHz.^19^ These radiofrequencies are able to penetrate deeply into dense and large-size products, resulting in even heating.^24,25^

In this study, we examined a solid-state MW heating technology as a possible method to dry Cannabis inflorescences rapidly and efficiently without deteriorating the quality of the resulting product. Solid-state MW allows for a high degree of control of the drying process relative to alternatives such as magnetron.^26^ We investigated how MW drying affects the composition of phytocannabinoids and terpenoids in medical Cannabis inflorescences.

## Materials and Methods

### Drying apparatus and operation method

The drying apparatus was a prototype based on a Miele oven Model H6800BM (Gütersloh, Germany) used for cooking that has been modified to eliminate the heating function. The apparatus combines an infrared (IR) sensor for Cannabis temperature measurement (Panasonic Grid-EYE^®^ AMG8833; Newark, NJ) and an RF module with a feedback control loop that was designed by GOJI (Kefar Sava, Israel). The RF module is controlled by an external computer (Lenovo T440, operation system—windows 7, I5, 4 GB RAM) with software developed by GOJI that displays the parameters relating to the drying process, including the forward power, reflected power, frequency, phase, energy absorption, and temperature. The module has a minimum and maximum frequency band operation of 2400 to 24,500 MHz, respectively, with a maximal transmitting power of 250 W. Dedicated shelves were designed and built for this oven to enable Cannabis drying in this prototype. A fan was included in the system to circulate the air and remove the moisture.

### Drying procedure

Inflorescences from a female predominant (−)-Δ^9^-*trans*-tetrahydrocannabinol (Δ^9^-THC) type I *Cannabis sativa* L. chemovar (Infiniti) were harvested at week 10 from flowering and either air dried at the Cronos Israel drying facility under controlled temperature and humidity conditions (16°C, 50% relative humidity) for 10 days or dried in the MW drying apparatus for the indicated hours. To determine the optimal drying temperature in the MW oven, samples of ∼100 g were dried at 40°C, 50°C, 60°C, and 80°C. The ambient conditions were 16°C and 50% relative humidity as well. The drying process was completed when samples were dry to the touch and the stem holding the inflorescences was broken easily, or when the drying time was greater than 4 h.

### Moisture measurement

The moisture content (dry basis) of the inflorescences was determined by the loss on drying method^27^ immediately after harvesting (fresh) and following the drying processes using a Mettler Toledo HB43 moisture analyzer (Agentek, Yakum, Israel). Briefly, the method used a drying temperature of 105°C, a standard heating profile, and an auto switch-off criterion (weight change of 1 mg within a 25-sec interval). The dried inflorescences were ground to a fine powder using an electric grinder and tested in triplicates.

### Water activity measurement

The water activity of Cannabis inflorescences (*n*=3) dried either traditionally or by MW drying at 50°C was measured using a Rotronic HygroPalm hygrometer (Bassersdorf, Switzerland).

### Scanning electron microscopy

Cannabis inflorescences after traditional drying or MW drying at 50°C were observed using scanning electron microscopy (SEM). The dried samples were sputter coated with Au-Pd alloy and then imaged using a JEOL JSM-IT200 SEM operated at an acceleration voltage of 20 kV.

### Chemical analysis of phytocannabinoids and terpenoids

Identification and quantification of phytocannabinoids were done by liquid chromatography methods.^8,28^ Terpenoid analysis was performed by gas chromatography/mass spectrometry (GC/MS) as described by Shapira et al.^29^ The results were normalized to the weight of the dry sample according to the moisture content. The full details are available in the Supplementary Data.

### Microbiological assay

Inflorescences immediately after harvesting and following traditional drying or MW drying at 50°C were sent to the Institute for Food Microbiology and Consumer Goods Ltd. (Nesher, Israel) for microbiological examination. The samples were tested for total combined yeast and mold count (TYMC) according to USP <61 > /Ph. Eur2.6.12. Microbial counts were expressed as colony-forming units per gram of sample (CFU/g).

### Statistical analyses

Statistical analyses were conducted using GraphPad Prism software version 9.0.0 (GraphPad, Inc.). A value of *p*≤0.05 was considered significant.

## Results

### Drying process

**FIG. 1.**
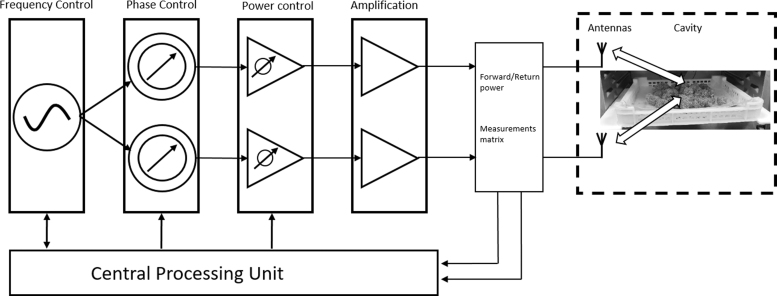
Schematic diagram of the solid-state microwave drying method. The signal generation unit generates MW signals with controlled frequencies. Each signal is split into two channels that go through a phase shifter and from there to a power amplification or attenuation that prepares the signal for final amplification by an amplifier unit of a constant gain. The energy is transmitted to the antennas in the cavity through each channel, and the returning energy from the cavity is measured through each antenna using the measurement matrix. Then, the central processing unit calculates the appropriate MW parameters, based on which it controls further signal generation, phase shift, and amplification. The process is iterative, so the frequency, phase, and amplitude of MWs transmitted to the Cannabis are controlled in a closed-loop until the end-point. The module controls the phase between the antennas in the range of 0 to 360 with ∼1.5° step. Of the total available FPCs, the module algorithm selects 6 different phase levels for each frequency. FPCs, frequency-phase combinations; MW, microwave.

Drying of Cannabis inflorescences with the solid-state MW oven was undertaken to compare the efficiency and quality of the MW-based drying method with the traditional drying method. The oven includes a specialized closed-loop system that uses multiple transmitter and receiver antennas to deliver drying energy to the sample load and collect reflections from it. A schematic diagram of the drying process is shown in Figure 1. The technology uses frequency sweep for the MW, which was set in the present study to a range from 2400 to 2500 MHz that is generated and amplified by solid-state sources and amplifiers. It can change ion migration and dipole rotation but does not lead to changes in molecular structure.^30,31^ In particular, the power transmission of each frequency is separately measured and controlled. The MWs are simultaneously transmitted through two transmitting antennas, under controlled and measured phase difference.

The system can handle multiple frequency-phase combination (FPC) states, and the total combinations can reach up to 30,000 states as the system uses up to 100 frequency points, and a phase resolution of a maximum of 300 phase points. The use of many FPC states increases the system's degrees of freedom, thus increasing the outcome uniformity, power efficiency, and detection ability. However, computational resources impact the required analysis calculation time, therefore, the system algorithm selects a subpart of the total available FPCs based on efficiency criteria as a compromise.

**FIG. 2.**
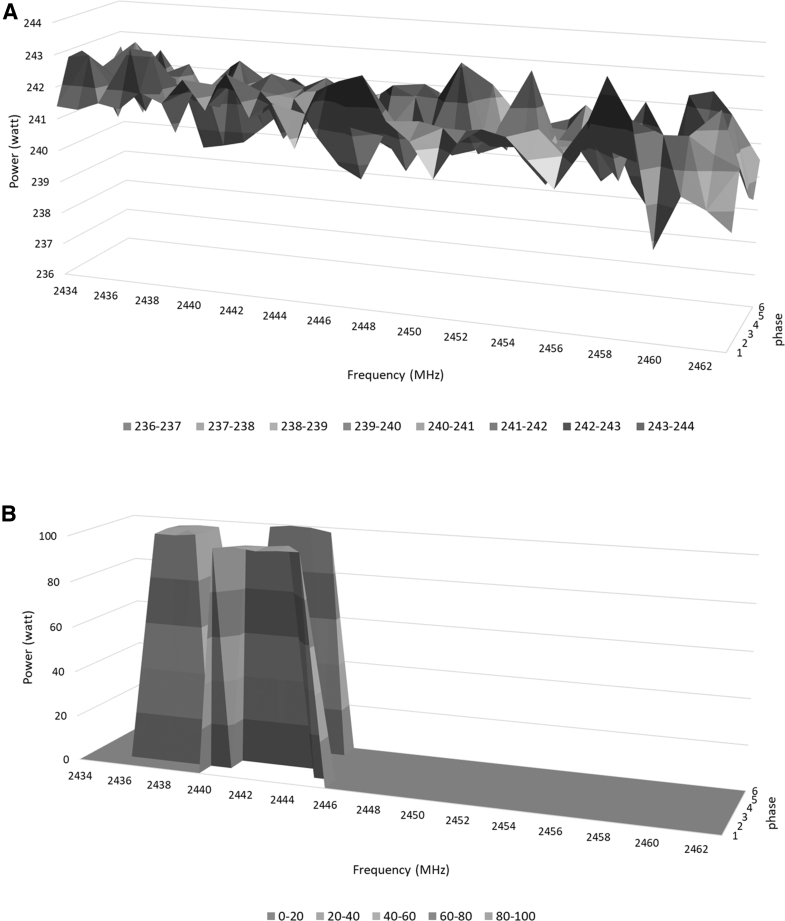
Forward-power envelope at various FPCs during the drying process. The forward-power envelope is displayed for a subset of the available frequencies at the beginning of the drying process **(**cycle 5, **A)** and after ∼25 min of drying **(**cycle 250, **B)**.

The transmission power at each FPC is controlled and measured during drying at each frequency and phase. These measurements are used together to estimate how much power is being absorbed by the Cannabis inflorescences at each FPC, and this information is used to adjust MW transmission in a closed loop. For the Cannabis drying, the power in the FPCs was adjusted so that a similar amount of energy was absorbed (not transmitted) at each of the FPCs. Notable, the absorption at each FPC changes during the drying, so the MW transmission protocol is constantly adjusted to maintain these conditions. In Figure 2, the forward power envelope measured during drying of Cannabis inflorescences is displayed at various FPC states. FPC represents the possible FPCs of the system at each moment during the drying period and is in correlation with the water content of the samples. In the beginning (Fig. 2A), most of the FPC states are involved in the drying process.

As the drying proceeds (Fig. 2B), the algorithm based on the parameters monitored during the drying process selects only a part of the FPC states to transmit, eliminating some and reducing power if needed. The main parameter is the transmitted FPC signal that results from the high moisture threshold criteria analyzed in the prior time window. In addition, the temperature of the inflorescences is constantly measured using IR thermometers that measure the temperature from a substantial portion of the surface area, and the power transmission is adjusted to ensure a constant temperature.

### Optimal drying temperature

To find the temperature of MW drying that best matches the evaporation of moisture by traditional drying, Cannabis inflorescences from a Δ^9^-THC-rich chemovar were dried in a traditional drying apparatus at 16°C and 50% relative humidity for 10 days and in the MW oven at various temperatures of either 40°C, 50°C, 60°C, or 80°C. Optimal drying conditions were defined as those only causing the smallest changes in the secondary metabolite composition relative to their composition before drying. The results were compared with those of the traditional drying process to evaluate the effectiveness and efficiency of the MW drying method.

The moisture content before drying and after the drying processes and the drying times are shown in Table 1. At a drying temperature of 40°C, the required drying time to reach the goal moisture content was greater than 4 h, therefore, the process was not completed before reaching sufficient moisture evaporation. On the contrary, at the higher drying temperatures, the drying time was shorter than 2 h and the moisture evaporation was substantial, resulting in final moisture content below 20%. As the temperature increases, the drying time required to achieve similar final moisture content decreases.

**Table 1.** Moisture Content and Drying Time for Cannabis Inflorescences Dried Traditionally or with the Microwave Oven at Different Temperatures

**Sample**	**Moisture before drying (%)**	**Drying time (DD: HH:MM)**	**Moisture after drying (%)**
Traditional	77.03±0.75	10:00:00	13.10±0.21
MW 40°C		00:04:15	25.96±0.47
MW 50°C		00:01:40	16.99±0.42
MW 60°C		00:00:50	16.33±0.02
MW 80°C		00:00:31	18.68±0.20

MW, microwave.

High temperatures were shown to affect the composition of cannabinoids in the Cannabis plant.^8,32,33^ Therefore, to determine the optimal drying temperature with the MW oven for Cannabis inflorescences in which the chemical cannabinoids' profile is best kept, changes in the secondary metabolite composition were assessed in comparison to traditional drying using high performance liquid chromatography with an ultraviolet detector (HPLC-UV).

**FIG. 3.**
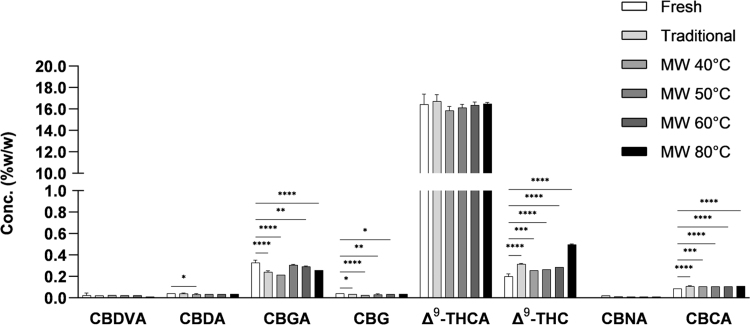
Concentrations of major phytocannabinoids in Cannabis inflorescences before and after drying traditionally or with the MW oven at different temperatures. Fresh—immediately after harvest, traditional drying—after 10 days at 16°C and 50% relative humidity, and MW drying—at the indicated temperatures. Data are reported as mean±SD of phytocannabinoid concentration (*n*=3, %w/w). Statistically significant differences between groups were calculated by one-way ANOVA (*p≤0.05, **p≤0.01, ***p≤0.001, ****p≤0.0001). The mean of each drying treatment was compared with the mean of the fresh material.

Figure 3 shows the concentration of some of the major phytocannabinoids in Cannabis inflorescences before and after the drying processes (among the 13 phytocannabinoids that were tested only those that were detected are shown in the figure). The full and abbreviated names of the phytocannabinoids identified can be found in the Supplementary Data. Phytocannabinoids are biosynthesized in their acidic form in the plant and undergo spontaneous decarboxylation with time under the influence of light or heat, resulting in the neutral form.^34^ We tested both the acidic and the neutral form of the phytocannabinoid. No unusual degradation products,^35^ such as isomerization (Δ^8^-THC), oxidation (CBN), or photodegradation (CBL) were observed.

Decarboxylation products, such as Δ^9^-THC and cannabigerol (CBG), were detected in all samples, including the fresh and traditionally dried samples. CBG concentration was very low and similar in all samples. The concentration of Δ^9^-THC, the product of Δ^9^-THCA decarboxylation, in the MW oven-dried samples was higher than in the fresh sample. However, in all samples dried at a temperature lower than 80°C, the concentration of Δ^9^-THC was similar to that of the sample dried traditionally. At a drying temperature of 80°C the concentration of Δ^9^-THC was significantly higher.

We confirmed the concentration of compounds that were detected by HPLC-UV and analyzed the concentrations of additional phytocannabinoids, including compounds with no available analytical standards, by ESI-LC/MS,^28^ and the comparison to traditional drying is presented in the Supplementary Data. The phytocannabinoid profiles were consistent with HPLC-UV results. No degradation products such as isomerization (Δ^8^-THC) or photodegradation (cannabielsoin/cannabielsoic acid/cannabicyclol) were found in all drying treatments. Oxidation products were detected at very low concentrations (cannabitriolic acid types and cannabinolic acid types), in concentrations similar to traditionally dried samples. Cannabinodiolic acid type oxidation products were not detected, but these are generally identified with high-cannabidiol (CBD) chemovars. As in the HPLC-UV results presented in Figure 3, decarboxylation products (Δ^9^-THC and CBG) were observed in all samples, including the fresh sample and the sample dried traditionally.

Other decarboxylation products were observed such as CBD and CBC. Δ^9^-THC and CBC concentrations, formed from decarboxylation of Δ^9^-THCA and cannabichromenic acid, respectively, were higher in the dried samples than in the fresh sample, including the traditionally dried sample. The sample dried at 80°C showed the highest concentration of decarboxylation products, as it coincides with previous studies.^8^

**FIG. 4.**
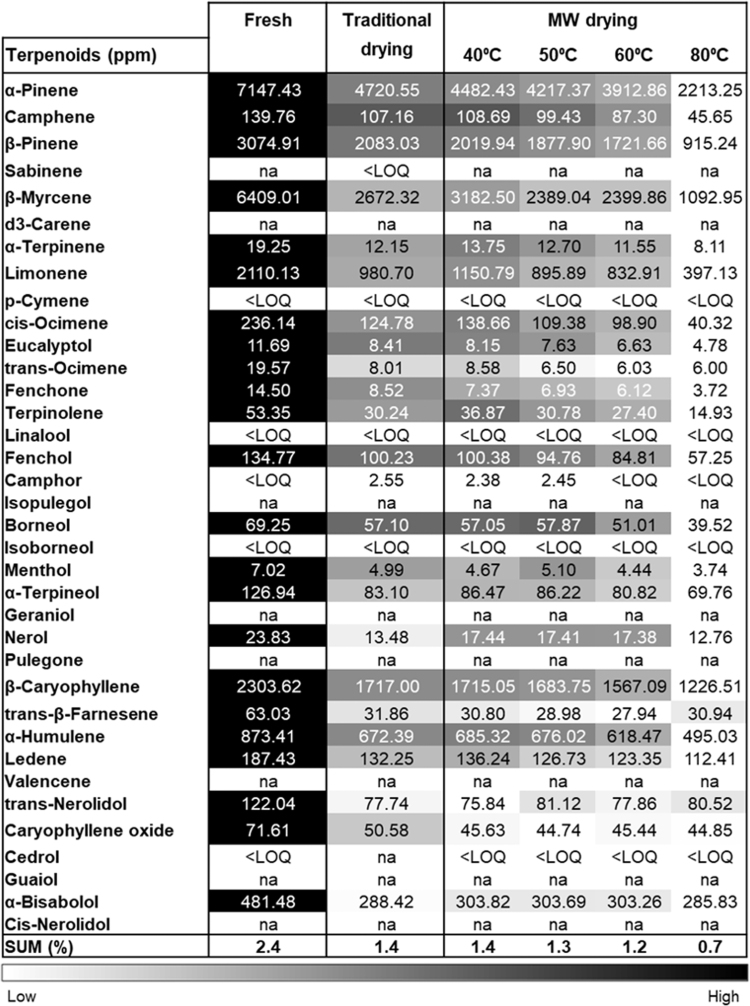
Heat map of terpenoid profile in Cannabis inflorescences before and after drying traditionally or with MW drying. The concentration of terpenoids (parts per million) was evaluated using GC/MS/MS for Cannabis Infiniti strain immediately after harvesting (fresh), after traditional drying, and after MW drying at the indicated temperatures. Absolute concentrations were color coded relative to the maximum value of each compound (*n*=2). SUM value represents the total terpenoid content in Cannabis inflorescences (%w/w). GC/MS, gas chromatography/mass spectrometry; LOQ, limit of quantification; na, not applicable.

Recognizing the potential for loss of volatile components at elevated temperatures,^29,36,37^ we further assessed the influence of MW drying on the terpenoid composition of Cannabis. Major terpenoids were analyzed in the fresh and dried samples (Fig. 4). Drying led to a decrease in the terpenoid concentration for all drying treatments, including the traditional drying process. In the case of the MW drying, the concentrations of terpenoids at 40°C and 50°C were relatively similar to that of traditional drying. The concentration of terpenoids after drying at 60°C was slightly lower relative to the two previous drying experiments at a lower temperature, however, still in the same range. A larger decrease and loss of terpenoids was observed at a drying temperature of 80°C, with values of almost half the concentration in the other drying trials. In addition, total terpenoid content in the inflorescences was analyzed. All the drying treatments decreased the terpenoid content, and more significantly as the drying temperature increased.

### Moisture assessment for drying termination end-point

**FIG. 5.**
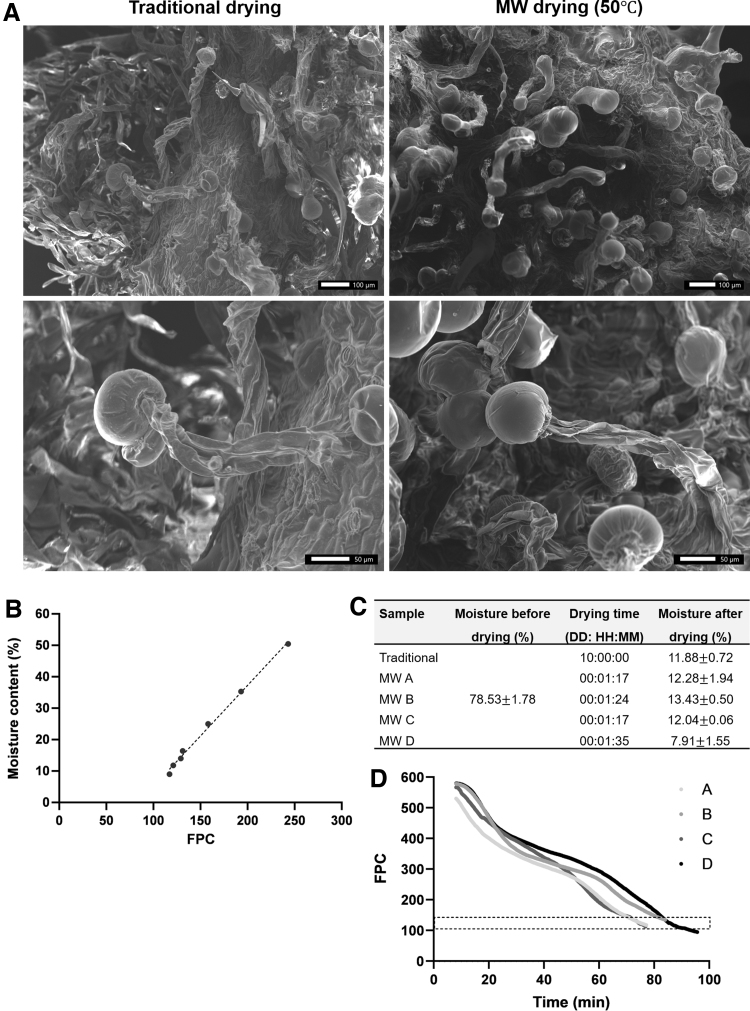
Prediction of drying process termination. **(A)** SEM images of Cannabis inflorescences dried either traditionally (left) or by MW drying at 50°C (right) and their corresponding magnified images (bottom). **(B)** Moisture content versus FPC number of Cannabis inflorescences dried with the MW oven at 50°C. The dotted line represents the linear regression. **(C)** Moisture content and drying time for Cannabis inflorescences dried with the MW oven at 50°C. (**A–D)** Four independent experiments. **(D)** FPC (normalized units) versus drying time in minutes. The dotted rectangle represents FPC numbers in the range of 0.50–0.53. Each FPC is characterized by an absorption efficiency value, and when the number of FPCs characterized by a value higher than a threshold crosses a predetermined number, the drying is stopped. SEM, scanning electron microscopy.

To assess the capability of the system to predict the moisture content of the samples, an empirical model developed by GOJI, correlating between the moisture content and the FPC states was examined. Different batches of inflorescences were dried at the chosen optimal temperature (50°C). The inflorescences from traditional and MW drying were visualized by SEM and no differences were seen in the structure of the trichomes (Fig. 5A). Next, samples of MW drying at 50°C were collected at predetermined time points and analyzed for moisture content. As seen in Figure 5B, a linear relationship (correlation coefficient of 0.996) was found between the moisture content and the FPC number. As expected, the FPC number decreases as the drying process progresses (Fig. 2).

This relationship was then applied to predict the termination of the drying process. Proper termination prevents overdrying or prolonging the drying time unnecessarily. An improper moisture content could result in buds that are too brittle and break apart, resulting in a lower-quality Cannabis product.^16^ Additional drying experiments were conducted at 50°C. The moisture content before drying and after the MW drying, and the drying times are shown in Figure 5C (four experiments were conducted, indicated by A–D). The drying end-point during these tests was determined according to the FPC value (Fig. 5D). The value of FPC decreases during the drying and when it reaches the range of 112–135 (dotted rectangle in Fig. 5D), the moisture content of the samples is about 12% (experiments A–C). In experiment D, the sample was allowed to dry beyond this threshold, resulting in final moisture content of about 8%.

Additionally, we tested the water activity of samples dried either traditionally or by MW drying at 50°C, and found it to be 0.495±0.002 and 0.571±0.002, respectively. Generally, to inactivate microbial activity, it is recommended to dry medical Cannabis to a maximum water activity level of 0.65 and moisture content value of 13%.^15,38^

As can be seen in Figure 5B, the drying time in these experiments was shorter than the time in the optimal drying temperature experiment at the same temperature of 50°C (Table 1) although the final moisture content was lower. This is likely because the experiment presented in Table 1 was performed at ambient conditions of 16°C and 50% relative humidity, and here the drying was performed at room temperature.

### The effect of MW drying on microbiological load in Cannabis inflorescences

**FIG. 6.**
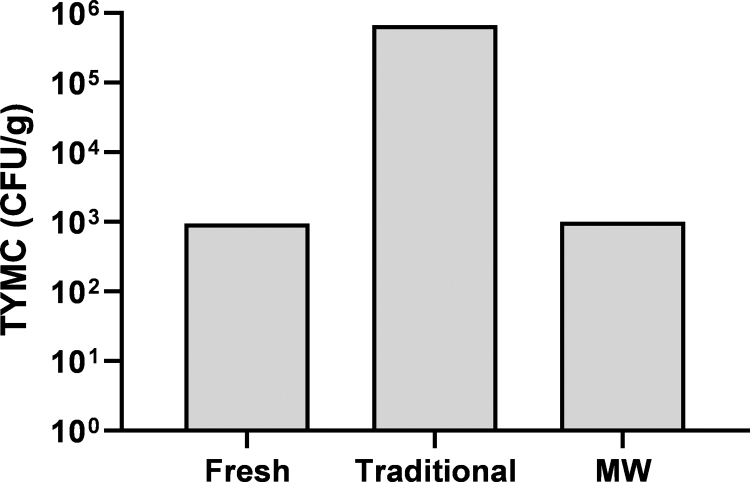
Microbiological assay of Cannabis inflorescences. TYMC of Cannabis Infiniti strain immediately after harvesting (fresh), after traditional drying, and after MW drying at 50°C. Microbial counts are expressed as colony-forming units per gram of inflorescence (CFU/g). TYMC, total combined yeast and mold count.

To assess the effect of the short drying time induced by this technology on the microbiological load of Cannabis inflorescences, we tested fresh or dried samples, either traditionally or in the MW oven (Fig. 6). Traditional drying resulted in a two-log increase in the TYMC. This is a known issue, as during the long drying time, water is available to microorganisms to promote growth.^12^ On the contrary, the TYMC after MW-based drying was similar to that of fresh inflorescences. The fast drying using this method reduces the water available to microbes, and therefore greatly assists in the prevention of microbial proliferation and spoilage.

## Discussion

There is a growing need for standardized and consistent high-quality Cannabis products. As traditional drying methods require an extended period of time and result in nonuniform drying, alternative methods are needed.

In this study, we found solid-state MW drying achieved similar moisture evaporation from Cannabis inflorescences in only a few hours, rather than several days with traditional drying. The solid-state technology allows for frequency control, which is practically impossible in magnetron technology used in conventional MW ovens, and power control, which is more subtle than allowed by commonly used magnetrons.^39^ Moreover, solid-state power amplifiers have the ability to provide feedback on the dynamic state of the processed material.^26^ This technology, therefore, produces a more uniform and consistent energy field during the heating process, resulting in an improved product in shorter times. Radiofrequencies excite and heat products internally, and not just the outer layers. The bulk heating effect produces uniform heating throughout the material, avoiding the large temperature gradient that occurs in conventional heating systems.^40,41^

As this method relies on heating and high temperatures were shown to affect the composition of cannabinoids in the plant,^8,32,33^ we evaluated the cannabinoid derivatives at different drying temperatures. At a drying temperature of 80°C, the concentration of Δ^9^-THC was high, probably due to the high temperature that promotes the heat-induced decarboxylation of Δ^9^-THCA into Δ^9^-THC.^8^ Of the different temperatures tested, the MW drying temperature that shortens the drying time substantially while retaining the phytocannabinoid composition of the Cannabis inflorescences was in the range of 50–60°C. For terpenoids, which are more volatile, at 40°C and 50°C their concentrations were relatively similar to that of traditional drying. Thus, the optimal drying temperature that significantly shortens the time while retaining the composition of the secondary metabolites was found to be 50°C.

During MW drying, the reflected energy is constantly collected and assessed, allowing the adjustment of the frequency and power parameters through the heating process. Since the dielectric characteristics of water change as a function of frequency and temperature,^42^ energy absorption during drying raises the temperature of the water and affects the reflected power, which enables the algorithm to select the relevant FPCs and estimate the plant moisture condition. The value of FPC provides a good indication of moisture content and the termination point of drying.

The significantly shorter drying time relative to traditional drying also reduces the potential risk of mold growth and pest contamination during the drying period, as was also previously reported.^13,43^ This is of major importance since Cannabis products, and especially products for medical purposes, are held to strict microbial specifications.^43,44^ However, although dried products are considered stable, certain pathogens, such as *Salmonella*, may still survive in low moisture environments upon sufficient rehydration.^45,46^

Importantly, scaling up is necessary for MW drying to be an eligible alternative for traditional drying, and the described technology can be scaled up to allow fast drying of large batches concurrently. The scaling up for industrial applications would focus on energy per mass. The overall energy of the system would be increased by increasing the number of transition sources and using an industrial conveyor capable of containing large masses.

## Conclusions

Solid-state MW drying is efficient and faster than traditional drying. The rapid removal of moisture shortens the drying process from days to hours and reduces the time available for microbial growth. At a drying temperature of 50°C, the chemical profile of phytocannabinoids and terpenoids was best kept to that of plants dried traditionally, suggesting less degradation by chemical reactions. Importantly, the moisture content assessment was achieved based on the FPC states of the system, allowing for the prediction of the drying termination end-point. Thus, MW drying provides an efficient and controlled post-harvest step for drying Cannabis inflorescences.

## Supplementary Material

Supplemental data

## Abbreviations Used

Δ9-THC: Δ9-tetrahydrocannabinol
CBC: cannabichromene
CBD: cannabidiol
CBG: cannabigerol
CBL: cannabicyclol
CBN: cannabinol
ESI-LC/MS: electrospray ionization liquid chromatography mass spectrometry
FPC: frequency-phase combination
GC/MS: gas chromatography/mass spectrometry
HPLC-UV: high performance liquid chromatography with an ultraviolet detector
IR: infrared
MW: microwave
RF: radiofrequency
SEM: scanning electron microscopy
THCA: tetrahydrocannabinolic acid
TYMC: total combined yeast and mold count
