# Supplementary material for: Solid-State Microwave Drying for Medical Cannabis Inflorescences: A Rapid and Controlled Alternative to Traditional Drying
Source: Cannabis Cannabinoid Res. 2024 Feb 12;9(1):397–408. doi: 10.1089/can.2022.0051 (PMC10874826; doi:10.1089/can.2022.0051)
Supplement: Supplemental data [file Supp_Data.docx]

Supplementary Data

**Solid-State Microwave Drying for Medical Cannabis Inflorescences - A Rapid and Controlled Alternative to Traditional Drying**

**Supplementary Methods**

**Figure S1.** Comparison of phytocannabinoid profiles of Cannabis inflorescences by ESI-LC/MS

**Table S1.** List of full and abbreviated names of phytocannabinoids

**Chemicals and reagents**

Liquid chromatography-mass spectrometric (LC/MS) grade acetonitrile, methanol, and water for the mobile phase; ethanol, for standard solutions and sample preparation; and headspace gas chromatography (GC) grade dimethyl sulfoxide (DMSO) were purchased from Mercury Scientific and Industrial Products Ltd. (Rosh Haayin, Israel). LC/MS grade acetic acid was obtained from BioLab Ltd. (Jerusalem, Israel); LC/MS grade n-Hexane for terpenoid standard solution preparation was purchased from Merck (KGaA, Germany). Phytocannabinoid analytical standards (> 98%) cannabigerol (CBG), (-)-Δ^9^-*trans*-tetrahydrocannabinol ($\Delta$^9^ -THC), cannabidiol (CBD), cannabichromene (CBC), cannabinol (CBN), cannabigerolic acid (CBGA), (-)-Δ^9^-tetrahydrocannabinolic acid ($\Delta$^9^ -THCA), cannabidiolic acid (CBDA), cannabinolic acid (CBNA), cannabichromenic acid (CBCA), (-)-$\Delta$^8^ -trans-tetrahydrocannabinol ($\Delta$^8^-THC), (-)-$\Delta$^9^ -tetrahydrocannabivarin ($\Delta$^9^ -THCV), cannabidivarin (CBDV), cannabidivarinic acid (CBDVA), and cannabicyclol (CBL) were purchased from Sigma-Aldrich (Rehovot, Israel); cannabichromevarin (CBCV) and cannabicitran were purchased from Cayman Chemical (Ann Arbor, MI, United States). Terpenoid analytical standards (> 95%), α-terpinene, linalool, fenchol, fenchone, menthol, borneol, α-terpineol, d3-Carene, p-cymene, camphor, isopulegol, isoborneol, geraniol, nerol, pulegone, trans-b-farnesene, cedrol, cis-Nerolidol, and caryophyllene oxide were purchased from Sigma-Aldrich (Rehovot, Israel); valencene (> 80% pure), α- and β-pinene, camphene, β-myrcene, α-bisabolol, β-caryophyllene, α-humulene, limonene, trans-nerolidol, cis and trans ocimene, and terpinolene (>90% pure) were purchased from Restek (PA, United States); eucalyptol, guaiol, and sabinene (>90% pure) were purchased from Extrasynthese (Genay, France); and ledene (>90% pure) was purchased from Toronto Research Chemicals (Toronto, Canada).

**Phytocannabinoid Identification and quantification**

Ground Cannabis inflorescences (100 mg) were accurately weighed in triplicates and extracted with 1 mL ethanol. Samples were agitated in an orbital shaker at 25°C for 15 min, and then centrifuged at 20,000 x g for 5 min. Prior to analysis, all samples were filtered through a 0.22 µm PTFE syringe filter (Lumitron ltd, Petah Tikva, Israel).

CBDVA, CBDV, CBDA, CBGA, CBG, CBD, THCV, CBCV, CBN, $\Delta$^9^-THC, $\Delta$^8^-THC, CBNA, CBL, CBC, THCA, CBCA, and cannabicitran were analyzed by ultra-high performance liquid chromatography with an ultraviolet detector (UHPLC/UV, Thermo Scientific, Bremen, Germany). Chromatographic separation was achieved using a HALO C18 Fused-Core column (2.7 µm, 150 × 2.1 mm), with a HALO guard column (2.7 µm, 5 x 2.1 mm), and a ternary A/B/C multistep gradient (solvent A: 0.1% acetic acid in water, solvent B: 0.1% acetic acid in acetonitrile, and solvent C: methanol). The multistep gradient program was established as follows: initial conditions were 50% B, which was gradually raised to 67% B over 3 min, held at 67% B for 5 min, and then raised to 90% B over the next 4 min, held at 90% B for 3 min, decreased to 50% B over the next 1 min, and finally held at 50% B for 4 min to re-equilibrate the system prior to the next injection. Solvent C was initially 5%, and was then lowered to 3% over the next 3 min, held at 3% for 5 min, raised to 5% over the next 4 min, and then kept constant at 5% throughout the run. Flow rate was 0.25 mL/min, column temperature was 30°C, and injection volume was 1 µL. Data acquisition was performed in full UV-Vis scan mode.

All other phytocannabinoids were identified and quantified using a similar UHPLC system coupled to a Q ExactiveTM Focus Hybrid Quadrupole-Orbitrap MS (Thermo Scientific, Bremen, Germany) and a similar chromatographic method as described above. Identification and absolute quantification of phytocannabinoids were performed by external calibrations. For electro spray ionization (ESI)-LC/MS analysis, the extracted samples were further diluted at ratios of 1:9, 1:99, and 1:999 v/v Cannabis extract to ethanol.

**Chemical analysis of terpenoids**

Ground Cannabis inflorescences samples (35 mg) were accurately weighed in duplicates and mixed with 100 µL LC/MS grade ethanol in 20 mL amber rounded bottom headspace vials sealed with a magnetic 32 mm PTFE septa cap. Terpenoids were analyzed using a Trace 1310 GC (Thermo Scientific, Germany) coupled to a TSQ 8000 Evo triple-quadrupole MS (Thermo Scientific, Germany), equipped with a DB-35MS UI capillary column (30 m × 0.25 mm x 0.25 µm, Agilent, United States). A CTC autosampler (Pal RTC, CTC Analytics, Switzerland) was used in SHS injection mode, with a headspace static tool in splitless mode. Terpenoid analysis was performed with external calibrations. All the results were normalized to the weight of the dry sample according to the moisture content.

**
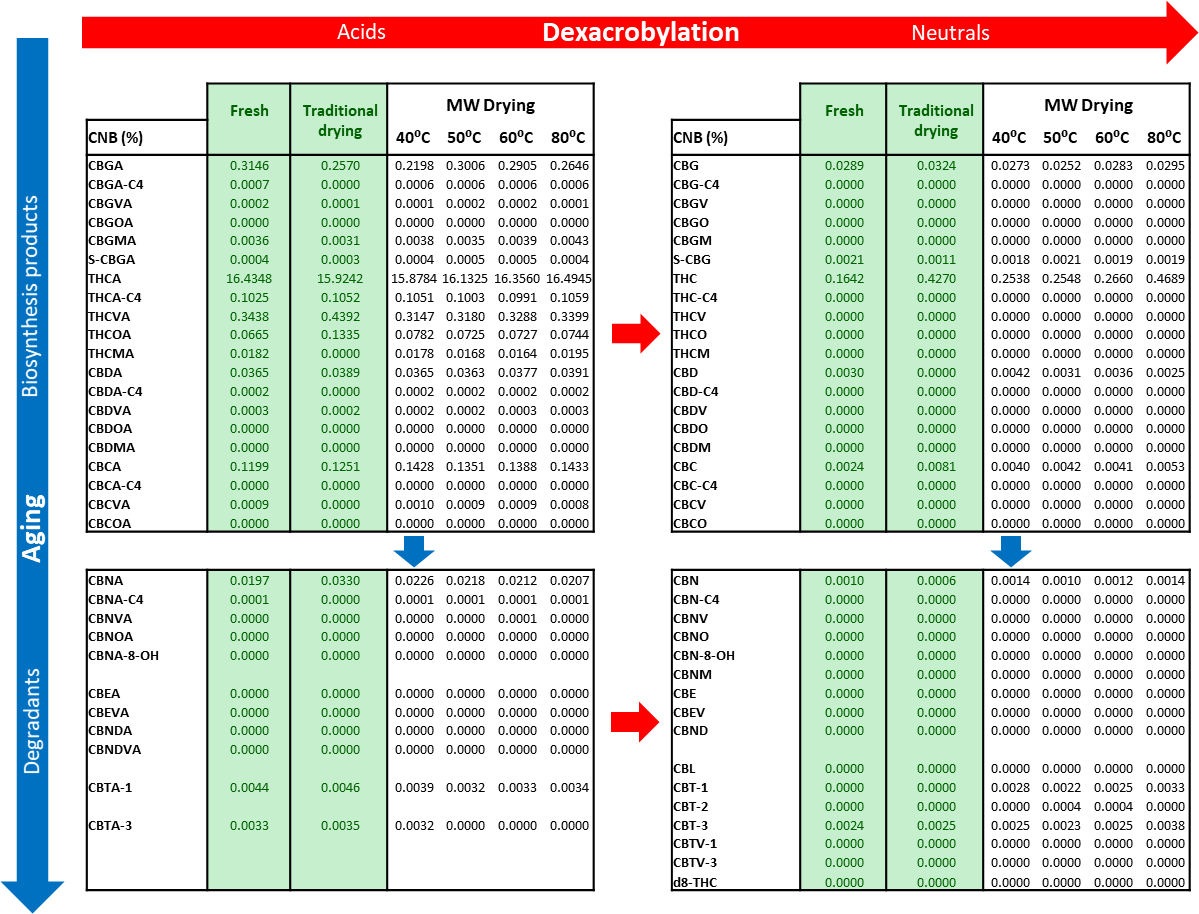

Figure S1.** **Comparison of phytocannabinoid profiles of Cannabis inflorescences by ESI-LC/MS.** The concentrations of phytocannabinoids were assessed by ESI-LC/MS after harvesting (fresh), traditional drying, and MW drying at different temperatures (n=1). Phytocannabinoids are arranged according to biosynthesis and degradation products. Red and blue arrows indicate decarboxylation and aging pathways, respectively.

**Table S1. List of full and abbreviated names of phytocannabinoids**

| **1. Cannabigerol (CBG) type** | **Abbreviated name** | **Acid Form** |
| --- | --- | --- |
| Cannabigerol-C5 | CBG | CBGA |
| Cannabigerobutol-C4^a^ | CBG-C4 / CBGB | CBGA-C4 |
| Cannabigerovarin-C3 | CBGV | CBGVA |
| Cannabigerorcol-C5 | CBGO | CBGOA |
| Sesquicannabigerol-C5 | S-CBG | S-CBGA |
| Cannabigerol monomethyl ether-C5 | CBGM | CBGMA |
| **2. Δ^9^-*trans*-tetrahydrocannabinol (Δ^9^-THC) type** | |  |
| (-)-Δ^9^-*trans*-Tetrahydrocannabinol-C5 | THC | THCA |
| (-)-Δ^9^*-trans*-tetrahydrocannabutol-C4 | THC-C4 / THCB | THCA-C4 |
| (-)-Δ^9^-*trans*-Tetrahydrocannabivarin-C3 | THCV | THCVA |
| (-)-Δ^9^-*trans*-Tetrahydrocannabiorcol-C1 | THCO | THCOA |
| (-)-Δ9-trans-Tetrahydrocannabinol monomethyl ether-C5 | THCM | THCMA |
| **3. Cannabidiol (CBD) type** |  |  |
| (-)-Cannabidiol-C5 | CBD | CBDA |
| (-)-Cannabidibutol-C4^a^ | CBD-C4 / CBDB | CBDA-C4 |
| (-)-Cannabidivarin-C3 | CBDV | CBDVA |
| (-)-Cannabidiorcol-C1 | CBDO | CBDOA |
| Cannabidiol monomethyl ether-C5 | CBDM | CBDMA |
| **4. Cannabichromene (CBC) type** |  |  |
| (±)-Cannabichromene-C5 | CBC | CBCA |
| (±)-Cannabibutol-C4^a^ | CBC-C4 / CBCB | CBCA-C4 |
| (±)-Cannabichromevarin-C3 | CBCV | CBCVA |
| (±)-Cannabiorchromorcol-C1 | CBCO | CBCOA |
| **5. Cannabinol (CBN) type** |  |  |
| Cannabinol-C5 | CBN | CBNA |
| Cannabibutol-C4^a^ | CBN-C4 / CBNB | CBNA-C4 |
| Cannabivarin-C3 | CBNV | CBNVA |
| Cannabiorcol-C1 | CBNO | CBNOA |
| Cannabinodivarin-C3 | CBNDV | CBNDVA |
| 8-hydroxycannabinol | CBN-8-OH | CBNA-8-OH |
| Cannabinol monomethyl ether-C5 | CBNM |  |
| **6. Δ^8^-trans-tetrahydrocannabinol (Δ^8^-THC) type** | |  |
| (-)-Δ^8^-*trans*-(6aR,10aR)-Tetrahydrocannabinol-C5 | Δ^8^-THC |  |
| **7. Cannabicyclol (CBL) type** |  |  |
| (±)-(1aS,3aR,8bR,8cR)-Cannabicyclol-C5 | CBL |  |
| **8. Cannabinodiol (CBND) type** |  |  |
| Cannabinodiol-C5 | CBND | CBNDA |
| **9. Cannabielsoin (CBE) type** |  |  |
| (5aS,6S,9R,9aR)-Cannabielsoin-C5 | CBE | CBEA |
| Cannabielsoin-C3 | CBEV | CBEVA |
| **10. Cannabitriol (CBT) type** |  |  |
| (±)-*cis/trans*-Cannabitriol-C5-1 | CBT-1 | CBTA-1 |
| (±)-*cis/trans*-Cannabitriol-C5-2 | CBT-2 |  |
| (±)-*cis/trans*-Cannabitriol-C5-3 | CBT-3 | CBTA-3 |
| Cannabitriol-C3-1 | CBTV-1 |  |
| Cannabitriol-C3-3 | CBTV-3 |  |
